# Insulin Injection Technique Education and Associated Knowledge Factors Among Physicians: Cross-Sectional Survey Study

**DOI:** 10.2196/65359

**Published:** 2025-12-08

**Authors:** Ida Ayu Made Kshanti, Nadya Magfira, Anak Agung Arie Widyastuti, Jerry Nasarudin, Marina Epriliawati, Md Ikhsan Mokoagow

**Affiliations:** 1 Division of Endocrinology, Metabolism and Diabetes, Department of Internal Medicine, Fatmawati General Hospital, South Jakarta, 12430, Indonesia, 62 81584149555; 2 Diabetes Integrated Care Center, Fatmawati General Hospital, Jakarta, Indonesia; 3 Indonesian Diabetes Educator Association, Jakarta, Indonesia; 4 Department of Internal Medicine, Faculty of Medicine, Universitas Indonesia, Jakarta, Indonesia; 5 Department of Internal Medicine, Fatmawati General Hospital, Jakarta, Indonesia

**Keywords:** insulin injection techniques, insulin therapy, diabetes management, health services, patient education, insulin education, outpatient treatment, therapy expert, awareness

## Abstract

**Background:**

Insulin therapy is crucial for managing type 2 diabetes mellitus, with its use steadily increasing in Indonesia and its effectiveness well established. However, prescribing insulin poses various challenges that can impact the effectiveness of insulin. Patient education is crucial for the successful implementation of insulin therapy. Proper insulin use remains insufficient in Indonesia.

**Objective:**

This study aims to investigate physicians’ knowledge and practice in providing education on insulin use to patients with type 2 diabetes mellitus in Indonesia.

**Methods:**

This study recruited potential participants (all physicians in Indonesia) through the internet using a convenience sampling method. The participants were asked to fill out a questionnaire. The questionnaire had 32 questions divided into 4 sections: demographics and clinical practice, practice of insulin education, the Indonesian insulin injection technique guideline, and knowledge of insulin injection techniques. The instrument used in this study was developed based on the *Pedoman Teknik Menyuntik Insulin Indonesia*, which was adapted from the international consensus by the Forum for Injection Technique and Therapy Expert Recommendations. The survey lasted from February 2021 to March 2021. Data were analyzed using the Kruskal-Wallis tests.

**Results:**

A total of 823 participants were included in the analysis. Out of 823 participants, 680 (82.6%) had given insulin education to patients at least once during the last 30 days. However, out of 823 participants, only 479 (58.2%) used specific guidelines in their practice, with only 280 (34.0%) aware of the Indonesian guidelines. Out of 823 participants, 815 (99.1%) agreed that insulin injection techniques would affect clinical results. The median score of knowledge about insulin injection techniques was 7 (IQR 2) among the study participants, indicating good knowledge. Profession was the only variable significantly associated with knowledge scores, with consultants in endocrinology, metabolism, and diabetes achieving the highest median scores, and other physicians the lowest (*P*<.001).

**Conclusions:**

Most physicians in this study reported providing education to their patients. However, there was still a gap between the guidelines and the practice of insulin education, as indicated by the lack of awareness and a fair level of knowledge about the Indonesian guidelines.

## Introduction

### Background

According to the International Diabetes Federation, Indonesia was ranked as the seventh country with the largest number of adults with type 2 diabetes mellitus in 2019 [1]. Among the 10.7 million individuals with type 2 diabetes mellitus, only 0.7% to 53% achieved their treatment targets, depending on the parameter used (either glycated hemoglobin [HbA_1c_] or fasting plasma glucose) [2-6]. Meanwhile, glycemic stability is needed to reduce the morbidity and mortality associated with type 2 diabetes mellitus [7]. To date, advances in knowledge and science have provided various approaches to achieving glycemic control. In addition to lifestyle modifications, oral and systemic antihyperglycemic agents help patients maintain their blood glucose levels. Insulin, one of the most used agents, is a crucial part of the treatment plan because it is recommended if oral antihyperglycemic agents fail to maintain glycemic stability. The Indonesian guideline on the management and prevention of type 2 diabetes mellitus recommends the use of insulin in cases of persisting HbA_1c_ level ≥7.5% following the use of 1 or 2 oral antihyperglycemic agents, HbA_1c_ level >9%, rapid weight loss, severe hyperglycemia with ketosis, hyperglycemic crisis, severe stress conditions (systemic infections, major surgery, acute myocardial infarct, and stroke), uncontrolled gestational diabetes mellitus, severe kidney or liver function disorders, contraindications and allergies of antihyperglycemic agents, and perioperative conditions as indicated by the clinicians. In Indonesia, insulin is mainly prescribed by internists. However, general practitioners (GPs) may also prescribe insulin for patients with type 2 diabetes mellitus, provided there are no complications [8-10].

With the growing availability and affordability of insulin, as well as improved access to health care services overall in the country, the use of insulin is expected to increase in the coming years [11]. Indonesia has adopted guidelines from the Forum for Injection Technique and Therapy Experts Recommendations for insulin injection techniques, titled *Pedoman Teknik Menyuntik Insulin Indonesia* (PTMII) 2017. The Indonesian guidelines provided recommendations for outpatient and inpatient settings, as well as special populations [12,13]. However, prescribing insulin to patients with type 2 diabetes mellitus is not without its challenges [14,15]. Previous studies showed that lack of access to care and medications, an inadequate health care system, and inadequate education have become obstacles to starting therapy [9,16,17].

In a preliminary cross-sectional study conducted at one of the tertiary hospitals in Jakarta, only 6.1% of 368 patients with type 2 diabetes mellitus received education while receiving outpatient treatment [18]. Similar findings have been reported in other countries. In the United States, type 2 diabetes mellitus education was reported to be received by only 52% of adult patients with type 2 diabetes mellitus. An Iranian qualitative study did not mention the exact number, but the health care professionals reported that most patients have a low level of type 2 diabetes mellitus education. These findings indicate that the patients’ education regarding type 2 diabetes mellitus is generally low, although the United States and Iran had better economic status than Indonesia [19,20]. Indonesia’s health care system, particularly its infrastructure, has become a significant barrier to type 2 diabetes mellitus management in general. A higher mortality rate is observed in government or military hospitals compared to private hospitals, indicating substantial disparities in health care infrastructures [21]. Education is another vital part because some Asians have skepticism toward Western therapy [9]. It is widely known that patients’ awareness and self-management are keys to successful type 2 diabetes mellitus care, especially for those receiving insulin. Thus, patient education and empowerment are essential responsibilities of health care professionals [22,23].

### Objectives

Moreover, there is still a significant gap between insulin injection practice and current guidelines [24]. A study by Chen et al [9] reported that GPs in the Asia Pacific regions, including Indonesia, felt a lack of support and confidence in administering insulin. To improve the outcomes of type 2 diabetes mellitus education, it is vital to examine these issues. To our knowledge, previous studies in Indonesia mostly focused on patients’ knowledge of type 2 diabetes mellitus and its impact on their self-management of diabetes. There is still a paucity of studies that have looked at physicians in general. Thus, this study aims to investigate the practice of insulin education and the knowledge of insulin injection techniques among physicians working in Indonesia.

## Methods

### Study Design

This is a cross-sectional web-based open survey in which data collection lasted for 2 weeks, from February 28, 2021, to March 14, 2021. The authors recruited potential participants (the target population consisted of all physicians in Indonesia) through convenience sampling by distributing invitations via WhatsApp groups for seminars, workshops, training sessions, university alumni networks, hospitals, and professional or academic societies. Our study used a targeted recruitment strategy to minimize concern regarding potential self-selection bias in web-based surveys. The survey link (Google Forms) was disseminated specifically to licensed physicians in Indonesia through professional networks, associations, and institutional mailing lists. Eligibility criteria were clearly stated in the informed consent section before accessing the questionnaire. Only physicians who prescribed insulin for patients with type 2 diabetes mellitus in the last 3 months were eligible to participate, as stated in the inclusion criteria. This inclusion criterion was determined to prevent the potential recall bias. Potential participants could then read a detailed explanation about the study on the first page of the survey, that is, study objectives, time needed to complete the questionnaire, and data anonymity, before they consented and proceeded to the questionnaire (Multimedia Appendix 1). In addition, the questionnaire included screening questions to verify eligibility before allowing completion. While anonymous and voluntary, this approach ensured that only qualified physicians participated in the study.

The questionnaire was developed by the research team at the Fatmawati Central General Hospital Integrated Diabetes Service Center, comprising 2 endocrinologists, 2 internists, and 1 epidemiologist. The questionnaire had 32 questions divided into 4 sections. The first section consisted of 9 questions regarding respondent demographics and clinical practice, covering age, professional background, workplace, years of experience, number of patients with type 2 diabetes mellitus treated, and insulin prescriptions issued in the last 30 days. Section 2 included 7 questions about the practice of insulin education, exploring the frequency of patient education, use of specific guidelines, awareness of national guidelines, and perception of responsibility in delivering insulin injection education. Section 3 consisted of 6 questions based on the adaptation of the Indonesian insulin injection technique guideline, assessed through 20 true or false statements derived directly from the PTMII. Topics included injection site selection, needle length and gauge, skinfold techniques, rotation, timing of injection, and hygiene procedures. The final section consisted of 10 questions about the knowledge of insulin injection technique. The knowledge is considered good if the participant achieves a score of 7 to 8 and very good if the participant achieves a score of 9 to 10. The instrument used in this study was developed based on the PTMII, which was adapted from the international consensus by the Forum for Injection Technique and Therapy Expert Recommendations [12]. This guideline is also endorsed by the Indonesian Society of Endocrinology.

The questionnaire was delivered across 7 online pages: an informed consent page, a screening questions page, and 4 pages covering each section, and a completeness check. The completeness check was to ensure that the participants had completed the questionnaire. If there were questions that had not been filled out, the participants could not submit the data. On the last page, participants were also able to review and change their answers before submitting the results.

The questionnaire was assessed for usability, technical functionality, and reliability before its final version was distributed to the study participants. The questionnaire was pretested on 30 individuals not included in the main study (10 GPs, 8 internal medicine residents, 8 internists, and 4 endocrinologists), which yielded a Kuder-Richardson 20 reliability coefficient of 0.70. The final version of the questionnaire was then distributed among groups.

Data analysis was performed using SPSS (version 20; IBM Corp). The authors did not conduct data imputation or missing data analysis in this study. Numerical data were presented as mean (SD) or median (range), while categorical data were presented as frequency tables or bar graphs. The distribution of numerical data was examined using skewness, kurtosis, and the Kolmogorov-Smirnov test for normality. Bivariate analyses were performed using the Kruskal-Wallis tests.

### Ethical Considerations

Ethics approval for this study was obtained from the ethics committee of Fatmawati Central General Hospital on February 26, 2021 (10/KEP/II/2021). The study was conducted according to the Declaration of Helsinki. Informed consent was obtained before accessing the questionnaire. All data were kept confidential and protected in online files, which can only be accessed by the investigators. The participants did not receive any compensation in the study.

## Results

### Characteristics of Study Participants

A total of 929 physicians participated in the survey. During the preliminary analysis, the authors decided to exclude 100 participants with incomplete answers and 6 participants because of data inconsistency, that is, claiming to prescribe insulin in the last 1 month in 1 question, while there were no patients with type 2 diabetes mellitus under care during the last 30 days. These 6 participants were excluded to prevent the possibility of recall bias or fraud. Thus, 823 participants were included in the final analysis. Table 1 presents the study participants’ characteristics.

Out of 823 participants, 362 (44.0%) belonged to the 30‐ to 40 years age group, while more than 300 (36.5%) participants were internists. Out of 823 participants, 29 (3.5%) had type 2 diabetes mellitus, while 482 (58.6%) had one or more family members with type 2 diabetes mellitus. Out of 823 participants, 366 (44.5%) worked on Java. Out of 823 participants, 584 (70.9%) have worked less than 10 years in the field.

In Table 1, it is shown that out of 823 participants, 688 (83.6%) worked at hospitals and 290 (35.2%) worked at tertiary care hospitals. Out of 823 participants, 452 (54.9%) had provided diabetes mellitus education services. Out of 823 participants, 387 (47.0%) treated 10 to 50 patients with type 2 diabetes mellitus during the last 30 days and gave 1 to 10 insulin prescriptions to their patients.

**Table 1.** Characteristics of study participants (N=823).

Characteristics	Participants, n (%)
Age (years)	
<30	131 (15.9)
30‐40	362 (44)
40‐50	154 (18.7)
50‐60	119 (14.5)
>60	57 (6.9)
Professional specialty	
General practitioner	206 (25)
Internal medicine resident	210 (25.5)
Internist	300 (36.5)
Consultant in endocrinology, metabolism, and diabetes	72 (8.8)
Others	35 (4.2)
Location in Indonesia (island)	
Java	366 (44.5)
Sumatra	128 (15.5)
Kalimantan	46 (5.6)
Sulawesi	208 (25.3)
Bali, NTB^a^, and NTT^b^	54 (6.6)
Papua	21 (2.5)
Association with individuals with diabetes mellitus	
Oneself	29 (3.5)
Friend	132 (16)
Family	482 (58.6)
None	180 (21.9)
Working experience (years)	
1‐5	299 (36.3)
6‐10	285 (34.6)
11‐15	109 (13.2)
16‐20	49 (6)
>20	81 (9.9)
Practice setting	
Private primary clinic	29 (3.5)
Private specialist clinic	11 (1.3)
Private practice	39 (4.8)
Primary health care center	56 (6.8)
Primary care hospital	35 (4.3)
Secondary care hospital (limited specialties)	195 (23.7)
Secondary care hospital (limited subspecialties)	168 (20.4)
Tertiary care hospital (top referral hospital)	290 (35.2)
Availability of type 2 diabetes mellitus education service	
Yes	452 (54.9)
No	371 (45.1)
Number of patients with type 2 diabetes mellitus treated in the last 30 days	
0	22 (2.7)
1‐10	235 (28.5)
10‐50	387 (47)
50‐100	102 (12.4)
>100	77 (9.4)
Number of insulin prescription in the last 30 days	
0	100 (12.2)
1‐10	359 (43.6)
10‐50	255 (31)
>50	109 (13.2)

a NTB: Nusa Tenggara Barat.

b NTT: Nusa Tenggara Timur.

### Insulin Education Practice Among Study Participants

Table 2 shows the practice of insulin education among study participants. Out of 823 participants, 680 (82.6%) claimed that they had given insulin education to patients, with 476 (57.8%) participants having given education 1 to 10 times during the last 30 days. Out of 823 participants, 514 (62.5%) said that physicians are responsible for educating patients. Only 4 (0.5%) out of 823 participants said that the responsibility should be given to other professionals. Out of 823 participants, 815 (99.0%) agreed that insulin injection techniques would affect clinical results, with 691 (84.0%) participants recommending the abdomen as the area for insulin injection. Out of 823 participants, 344 (41.8%) did not follow specific guidelines when educating their patients.

Out of 823 participants, 280 (34%) were aware of the Indonesian guidelines for insulin injection techniques. In total, 247 (88.2%) out of the 280 participants used the guideline in their practices. Awareness of the guideline mostly came from societies, such as the Study Society for Diabetes, the Society of Diabetes Educators, and the Society of Endocrinologists (169/280, 60.4%), as well as seminars or symposia (109/280, 38.9%). Out of the 280 participants, 2 (0.7%) learned about the guidelines during their residency.

**Table 2.** Insulin education practice (N=823).

Practice of insulin education	Participants, n (%)
Have you ever given insulin education to patients	
Never	143 (17.4)
Ever	680 (82.6)
Frequency of insulin education in the last 30 days	
0	149 (18.1)
1‐10	476 (57.8)
10‐50	170 (20.7)
>50	28 (3.4)
Use of specific guideline	
Yes	479 (58.2)
No	344 (41.8)
Recommended area for injection	
Abdomen	691 (84)
Upper arm	101 (12.3)
Thigh	25 (3.0)
All	5 (0.6)
Others	1 (0.1)
Which health care professional should be responsible for educating patients	
Pharmacists	30 (3.6)
Physicians	514 (62.5)
Diabetes educators	229 (27.8)
Nurses	46 (5.6)
Other professionals	4 (0.5)
Insulin injection technique would affect clinical results	
Agree	815 (99.0)
Disagree	8 (1.0)

### Awareness of Important Aspects of Insulin Injections Across Professions

Table 3 shows what participants thought was the most important aspect of the insulin pen injection. Out of 823 participants, 454 (55.2%) regarded the area of injection as the most important, whereas 51 (6.1%) regarded the needle gauge as the most vital aspect of insulin injection.

**Table 3.** Awareness of important aspects of insulin pen injection across professions (N=823).

Professional specialty	Needle length, n (%)	Area of injection, n (%)	Disposable needle, n (%)	Skinfold pinching, n (%)	Size of needle gauge, n (%)	Subtotal, n (%)
General practitioner	14 (18.4)	106 (23.3)	38 (30.9)	36 (30.3)	12 (23.5)	206 (25.0)
Internal medicine resident	16 (21.1)	121 (26.7)	28 (22.8)	32 (26.9)	13 (25.5)	210 (25.5)
Internist	29 (38.2)	166 (36.6)	45 (36.6)	42 (35.3)	18 (35.3)	300 (36.5)
Consultant in endocrinology, metabolism, and diabetes	7 (9.2)	46 (10.1)	9 (7.4)	5 (4.2)	5 (9.7)	72 (8.8)
Others	10 (13.1)	15 (3.3)	3 (2.4)	4 (3.4)	3 (5.9)	35 (4.2)

### Knowledge About Insulin Injection Technique

Table 4 lists the true or false questions on knowledge about insulin injection techniques. Questions in which the majority of participants (737/823, 89.6%) answered correctly were “Insulin absorption is influenced by the correct choice of injection area” and “The angle of insulin injection is usually perpendicular to the area of injection.” Meanwhile, the questions with the most incorrect answers were “Before injecting insulin at home, patients must always disinfect with alcohol” (244/823, 29.6%) and “The fastest-absorbing location for insulin injection is the abdominal area” (256/823, 30.6%).

**Table 4.** Questions regarding knowledge about insulin injection techniques (N=823).

Questions	True or false	Correct answer, n (%)
Insulin absorption is influenced by the correct choice of injection area	True	737 (89.6)
The rate of insulin absorption is constant regardless of the injection area	False	640 (77.8)
Injection time of rapid-acting insulin analogs (Aspart, Glulisin, and Lispro) is the same as that of short-acting human insulin (Actrapid and Humulin-R)	False	594 (72.2)
Injecting insulin on intramuscular tissue may relieve the pain better than subcutaneous tissue	False	577 (70.1)
Injecting rapid-acting insulin analogs (Aspart, Glulisine, and Lispro) on thigh areas is good for blood glucose control	False	475 (57.7)
Removing the needle within less than 10 seconds after injecting insulin can reduce the amount of insulin injected	True	672 (81.6)
The fastest-absorbing location for insulin injection is the abdominal area	False	252 (30.6)
The best location for insulin injection is areas with lipohypertrophy	False	605 (73.5)
The angle of insulin injection is usually perpendicular to the area of injection	True	737 (89.6)
Before injecting insulin at home, patients must always disinfect with alcohol	False	244 (29.6)

Regarding the participants’ scores on knowledge about insulin injection techniques, many participants obtained a total score of 6 and 8, that is, 22% (181/823) and 21% (173/823), respectively. Out of 823 participants, the lowest score of 2 was achieved by 6 (0.7%), while 22 (2.7%) attained a perfect score of 10. The median score was 7 (IQR 2), indicating good knowledge.

Table 5 shows the median knowledge scores about insulin across demographic characteristics. Professional specialty was the only variable significantly associated with knowledge scores, with consultants in endocrinology, metabolism, and diabetes achieving the highest median scores, and other physicians and GPs the lowest.

**Table 5.** Knowledge scores across demographic characteristics (N=823).

Demographic characteristics	Score, median (range)	*P* value
Age (y)		.05
<30	6 (3-9)	
30‐40	7 (2-10)	
40‐50	7 (2-10)	
50‐60	7 (2-10)	
>60	7 (2-10)	
Professional specialty		<.001
General practitioner	6 (2-10)	
Internal medicine resident	7 (3-10)	
Internist	7 (2-10)	
Consultant in endocrinology, metabolism, and diabetes	8 (5-10)	
Others	6 (2-10)	
Location in Indonesia (island)		.12
Java	7 (2-10)	
Sumatra	7 (2-9)	
Kalimantan	7 (3-10)	
Sulawesi	7 (3-10)	
Bali, NTB^a^, and NTT^b^	7 (3-10)	
Papua	6 (3-10)	
Close association with individuals with diabetes mellitus		.94
Oneself	7 (4-9)	
Friend	7 (2-10)	
Family	7 (2-10)	
None	7 (3-10)	
Working experience (y)		.28
1‐5	7 (2-10)	
6‐10	7 (3-10)	
11‐15	7 (2-10)	
16‐20	6 (3-10)	
>20	6 (2-10)	

a NTB: Nusa Tenggara Barat.

b NTT: Nusa Tenggara Timur.

## Discussion

### Principal Findings

This study examined the practice of insulin education and knowledge about insulin injection techniques among physicians working in Indonesia. To the authors’ best knowledge, the most recent similar study was conducted by a pharmaceutical company in 2012 [6]. Given the numerous challenges surrounding the management of type 2 diabetes mellitus in Indonesia, this study aimed to address the research gap and build on the evidence to improve outcomes for type 2 diabetes mellitus care. More than half of the participants were aged ≤40 years. This was supported by the fact that the majority of them were GPs, internal medicine residents, or internists.

It seemed that participants in this study regarded insulin education as important because almost all of them had given insulin education to patients in the last 30 days. This was supported by another finding that 99% of them agreed that the insulin injection technique would affect clinical results, and more than 80% of the participants thought that the responsibility for patient education lies with physicians and diabetes educators. However, more than 40% of the participants did not use specific guidelines in their insulin education practices. This may be due to the finding that 66.98% of the participants were not aware of the Indonesian guideline for insulin injection techniques (PTMII). Participants who were aware of the guidelines mainly received the information through studies or professional societies and seminars or symposia. This finding underscores the importance of further training or education and networking through societies to update knowledge and practices in type 2 diabetes mellitus management. Participants who were not aware of the guideline further stated that they would appreciate it if the study or professional societies could provide webinars or sessions to disseminate the guideline. The distribution of guidelines through social media, such as WhatsApp groups, was also encouraged by many participants.

While participants in this study were generally aware of the importance of the area of insulin injection, knowledge of needle length and size of needle gauge remained low, as only a small proportion of the participants (<10%) regarded them as the most important aspects of insulin injection. The results of this study also showed that the majority of the participants had a good knowledge of insulin injection techniques from the Indonesian guideline PTMII, as evidenced by a median score of 7 (IQR 2). In this study, age, workplace, personal or family history of diabetes mellitus, and years of working experience were not associated with knowledge scores. These findings are notable, as they differ from those reported in other studies.

### Comparison With Prior Work

Examining the geographical distribution of the participants, the pattern matched that of the general population in Indonesia, with a majority concentrated on the island of Java [24]. Thus, it can be assumed that the participants in this study were representative of the population of physicians in Indonesia. While no similar study assessed the compliance of physicians toward insulin injection guidelines, a review emphasizes that there are always barriers between physicians and practice guidelines. These barriers include established personal routines, the complexity of skills required, and physician resistance [14].

Although needle length does not directly affect glycemic control, it is important for correct insulin deposition (subcutaneous vs intramuscular) because there is a greater risk of intramuscular injections with longer needles [25]. Moreover, longer and larger needles would cause more pain and, therefore, make patients uncomfortable during injection [26]. The use of disposable needles must also be encouraged to reduce local complications, such as lipohypertrophy [27,28].

Physicians in Iran also demonstrated fair knowledge of insulin injection techniques (66.29% correct answers), which was higher than in Egypt (where 57% lacked knowledge) but lower than in Singapore (median score 8) [29-31]. However, these findings are not directly comparable due to differences in the knowledge content tested, scoring methods, or cutoff points for categories (poor, fair, and good), and medical curricula across countries. Nonetheless, there is a need to improve Indonesian physicians’ knowledge about injection site selection and disinfection procedures, as most participants answered these questions incorrectly.

One finding that was consistent with other studies was that consultants tended to score better than junior physicians [29,31-33]. This may not be surprising as consultants, in general, gain deeper knowledge through further formal training. However, it also indicates that physicians in Indonesia, especially GPs, still need to participate in more continuing medical education programs to increase their knowledge about type 2 diabetes mellitus care, particularly regarding insulin injection techniques.

Age was found to be associated with knowledge among physicians in Iran and Egypt; however, this may be caused by the categorization of age into 2 groups only, with a cutoff age of 40 and 32 years, respectively [29,31]. Meanwhile, in this study, there was no strong association between age and knowledge when age was categorized into 5 groups. Another interesting finding in this study was that only physicians aged <30 years had different ranges of scores; that is, they had lower median scores, better minimum scores, and lower maximum scores. This could be due to physicians aged <30 years being generally not yet in the fellowship (consultant) stage of training and thus lacking the knowledge needed to score well. This finding could also explain why there would be an association between age and knowledge when the cutoff point is set around that number.

Studies in Iran and Egypt found an association between workplace location and knowledge, which differs from the findings of this study [29,31]. This may be due to the different operational definitions used in each study. In this study, working location was defined by the main islands where the physicians practiced, while the Egypt study categorized it as rural versus urban and the Iran study considered whether it was a teaching hospital [29,31]. Looking at the results of this study alone, it is rather commendable that there was no significant difference in knowledge between physicians working in Java and outside Java, considering the common assumption that most of the country’s development and growth are concentrated in Java.

Personal and family history of diabetes mellitus was not found to be associated with knowledge in this study, which was in contrast to the studies conducted in Iran and Egypt [29,31]. However, this may be because those factors were combined into a single variable in this study, whereas they were treated as separate variables in the other studies. Working experience was also not associated with knowledge, consistent with the study in Egypt, where years of experience influenced physicians’ attitudes in practice but not their knowledge [29].

### Strengths and Limitations

A key strength of this study is that it is one of the few to investigate the knowledge and practice of insulin education among physicians in Indonesia. Thus, the results of this study could provide the basis for future research on similar topics. However, this study has several limitations. First, due to the amount of missing data, the study results must be interpreted accordingly. Second, due to the open and anonymous nature of the online survey and wide dissemination strategy, the exact number of invitations sent could not be determined; therefore, a formal response rate could not be calculated. We acknowledge this as a limitation, common to large-scale online surveys. Due to the anonymous nature of the survey, we were unable to identify or contact the participants directly. Hence, we tried to reach as many potential participants as possible to avoid a low sample size. Third, we also acknowledge that the absence of technical constraints on duplicate submissions is a limitation of this convenience sampling approach, common in anonymous online surveys. Due to the anonymous nature of the survey, we could not identify the participants. Hence, future studies should develop strategies to avoid this, such as filtering by IP address or restricting each email address to a single response. Fourth, there might be a recall bias due to the nature of this research. However, we tried to limit the study participants who had been prescribed insulin in the past 3 months and excluded those with inconsistent answers to avoid this. Fifth, as the questionnaire was newly developed, there may be a question about its validity. Usability, technical functionality, and reliability tests were conducted before the final version was distributed to the study participants. This was to ensure its validity and reliability. Sixth, there might be a selection bias due to the eligibility for prescribing insulin. However, we ensured that we reached all different subject groups for this study, including GPs; internists; consultants in endocrinology, metabolism, and diabetes; and other physicians.

### Future Directions

Future research should be conducted to explore the barriers and facilitators to diabetes mellitus or insulin education from the perspectives of both patients and physicians. In response to the demands from participants, it would be helpful if the study and professional societies in Indonesia could increase the promotion and dissemination of the guideline by delivering webinars or sharing the guideline through social media. Seminars should focus on aspects of insulin injection techniques that Indonesian physicians find difficult, such as the areas of injection and sterilization procedures, as well as aspects that physicians were least aware of, that is, needle length and size of needle gauge. Finally, physicians in Indonesia, especially GPs, would benefit from participating in more continuing medical education programs related to type 2 diabetes mellitus care and insulin injection techniques to improve patient outcomes.

### Conclusions

In conclusion, most physicians in this study stated that they had provided education to their patients in the last 30 days. Moreover, there was still a gap between the guidelines and practice of insulin education, as indicated by the lack of awareness and a fair level of knowledge about the Indonesian insulin injection technique guideline.

## Supplementary material

10.2196/65359 Multimedia Appendix 1 Questionnaire.

10.2196/65359 Checklist 1 CHERRIES checklist.

## Abbreviations

GP: general practitioner
HbA_1c_: glycated hemoglobin
PTMII: *Pedoman Teknik Menyuntik Insulin Indonesia*

## References

[1] (2020). National diabetes statistics report 2020: estimates of diabetes and its burden in the United States. Centers for Disease Control and Prevention.

[2] Soewondo P, Ferrario A, Tahapary DL (2013). Challenges in diabetes management in Indonesia: a literature review. Global Health.

[3] Soewondo P, Soegondo S, Suastika K, Pranoto A, Soeatmadji DW, Tjokroprawiro A (2010). The DiabCare Asia 2008 study – outcomes on control and complications of type 2 diabetic patients in Indonesia. Med J Indones.

[4] Yuliastuti C, Arini D, Sari MP (2019). The control of diabetes mellitus in coastal communities in Surabaya region. KEMAS.

[5] Fountaine T, Lembong J, Nair R, Süssmuth-Dyckerhoff C (2016). Tackling Indonesia’s diabetes challenge: eight approaches from around the world. McKinsey & Company.

[6] Soetedjo NN, McAllister SM, Ugarte-Gil C (2018). Disease characteristics and treatment of patients with diabetes mellitus attending government health services in Indonesia, Peru, Romania and South Africa. Trop Med Int Health.

[7] Swinnen SG, Hoekstra JB, DeVries JH (2009). Insulin therapy for type 2 diabetes. Diabetes Care.

[8] (2021). Pedoman pengelolaan dan pencegahan diabetes melitus tipe 2 di Indonesia 2021 [Web page in Indonesian]. PP Perkeni.

[9] Chen R, Aamir AH, Feroz Amin M (2024). Barriers to the use of insulin therapy and potential solutions: a narrative review of perspectives from the Asia-Pacific region. Diabetes Ther.

[10] Allen-Taylor M, Ryan L, Winkley K, Upsher R (2022). Exploring the experiences and perspectives of insulin therapy in type 2 diabetes via web-based UK diabetes health forums: qualitative thematic analysis of threads. JMIR Diabetes.

[11] Ewen M, Joosse HJ, Beran D, Laing R (2019). Insulin prices, availability and affordability in 13 low-income and middle-income countries. BMJ Glob Health.

[12] (2021). Pedoman petunjuk praktis terapi insulin pada pasien diabetes mellitus 2021 [Web page in Indonesian]. PP Perkeni.

[13] Tandon N, Kalra S, Balhara YP (2017). Forum for Injection Technique and Therapy Expert Recommendations, India: the Indian recommendations for best practice in insulin injection technique, 2017. Indian J Endocrinol Metab.

[14] Barth JH, Misra S, Aakre KM (2016). Why are clinical practice guidelines not followed?. Clin Chem Lab Med.

[15] Simon AC, Gude WT, Holleman F, Hoekstra JB, Peek N (2014). Diabetes patients’ experiences with the implementation of insulin therapy and their perceptions of computer-assisted self-management systems for insulin therapy. J Med Internet Res.

[16] Adhikari M, Devkota HR, Cesuroglu T (2021). Barriers to and facilitators of diabetes self-management practices in Rupandehi, Nepal- multiple stakeholders’ perspective. BMC Public Health.

[17] Preechasuk L, Sriussadaporn P, Likitmaskul S (2019). The obstacles to diabetes self-management education and support from healthcare professionals’ perspectives: a nationwide survey. Diabetes Metab Syndr Obes.

[18] Kshanti IA, Soebijanto N, Epriliawati M, Mokoaogow MI (2018). Diabetic Foot Self-Care Practice among Patients with Diabetes Mellitus Type 2 in Fatmawati General Hospital.

[19] Abazari P, Vanaki Z, Mohammadi E, Amini M (2012). Inadequate investment on management of diabetes education. J Res Med Sci.

[20] (2024). Diabetes education linked to better care. Centers for Disease Control and Prevention.

[21] Darmawan ES, Permanasari VY, Nisrina LV, Kusuma D, Hasibuan SR, Widyasanti N (2024). Behind the hospital ward: in-hospital mortality of type 2 diabetes mellitus patients in Indonesia (analysis of national health insurance claim sample data). Int J Environ Res Public Health.

[22] McDuffie RH, Struck L, Burshell A (2001). Empowerment for diabetes management: integrating true self-management into the medical treatment and management of diabetes mellitus. Ochsner J.

[23] Nazar CMJ, Bojerenu MM, Safdar M, Marwat J (2016). Effectiveness of diabetes education and awareness of diabetes mellitus in combating diabetes in the United Kigdom; a literature review. J Nephropharmacol.

[24] (2022). Share of population in Indonesia in 2020, by main island. Statista.

[25] Hirsch LJ, Strauss KW (2019). The injection technique factor: what you don’t know or teach can make a difference. Clin Diabetes.

[26] Kalra S, Pathan F, Kshanti IA (2023). Optimising insulin injection techniques to improve diabetes outcomes. Diabetes Ther.

[27] Sharma M, Kumar R, Rohilla L, Angrup A, Yadav J, Dayal D (2022). Effect of reuse of insulin needle on glycaemic control and related complications in children with type 1 diabetes mellitus: a prospective observational study. Indian J Endocrinol Metab.

[28] Bandari E, Beuzen T, Habashy L (2022). Machine learning decision support for detecting lipohypertrophy with bedside ultrasound: proof-of-concept study. JMIR Form Res.

[29] Alemam DS (2022). Knowledge, attitude and practices of primary care physicians towards diabetes diagnosis and management in Damietta District –Egypt. The Egypt J of Community Medicine.

[30] Lee MKS, Liu Z, Quek TPL, Chew DEK (2013). Insulin-Related Knowledge Among Health Care Professionals at a Tertiary Hospital. Diabetes Spectr.

[31] Niroomand M, Ghasemi SN, Karimi-Sari H, Khosravi MH (2017). Knowledge, attitude, and practice of Iranian internists regarding diabetes: a cross sectional study. Diabetes Metab J.

[32] Alsaleem MA, Aldarbi MA, Alsaleem SA, Alsamghan AS (2018). The variance of knowledge and practices about diabetes mellitus in primary health care physicians of Jazan region, Kingdom of Saudi Arabia. Biomed Res.

[33] Bain A, Kavanagh S, McCarthy S, Babar Z (2019). Assessment of insulin-related knowledge among healthcare professionals in a large teaching hospital in the United Kingdom. Pharmacy (Basel).

