# Supplementary material for: Insulin Injection Technique Education and Associated Knowledge Factors Among Physicians: Cross-Sectional Survey Study
Source: JMIR Diabetes. 2025 Dec 8;10:e65359. doi: 10.2196/65359 (PMC12685283; doi:10.2196/65359)
Supplement: Multimedia Appendix 1 [file diabetes-v10-e65359-s001.pdf]

Thank you for opening this questionnaire

Indonesia is the country with the 7th largest population of diabetes mellitus in the world. However, only a few patients have controlled blood sugar levels. Education related to diabetes, including the use of insulin, is one of the pillars of diabetes management that greatly affects the success of therapy. However, how health workers conduct education, especially the use of insulin for diabetes, has never been evaluated.

The research team at the Integrated Diabetes Service Center (PLDT) of Fatmawati General Hospital, Jakarta, conducted a survey via the Internet on " Insulin Injection Technique Education and Associated Factors of Knowledge: A cross-sectional survey on knowledge and practice of insulin injection technique education among physicians in Indonesia"

This survey aims to determine the knowledge and practice of doctors in Indonesia in educating insulin injection techniques for diabetes mellitus patients.
The survey was filled out without listing your name (anonymous) and your answers will remain confidential.
The survey results will be published in forums and/or scientific journals.

Who can take this survey? All doctors who have prescribed insulin in the past month can take this survey.

If you need further explanation, contact: dr. Nadya Magfira, M.Epid (Tel. No.: 081285160994, e-mail: nadyamagfira@gmail.com)

Please “click” the link below if you agree to take the survey.

Thank you for your willingness to be part of this survey.
May you always be healthy.
APPENDIX 2.
QUESTIONNAIRE 1

Demographic data

Name	:	
Date of Birth	:	
Address	:	
Last educational background	:	
Year of graduation	:	

Work history

Curren work place:

- ❑ Municipal health center/ Clinic/ Private practice
- ❑ Type D/ C/ B hospital
- ❑ Type A hospital/ university hospital

Is there a diabetes education service for patients in your current workplace??

- ❑ Yes
- ❑ No

History of exposure to diabetes

Number of diabetic patients you treated in 1 month:

- ❑ Never
- ❑ 1-10
- ❑ 10-50
- ❑ 50-100
- ❑ > 100

People with diabetes around you:

- ❑ I have diabetes
- ❑ my family (living in the same house) has diabetes
- ❑ my family (not living in the same house) has diabetes
- ❑ my friend has diabetes
- ❑ None

How often do you prescribe insulin to patients in 1 month:

- ❑ Never
- ❑ 1-10

- ❑ 10-50
- ❑ > 50

Have you ever provided education on insulin injection techniques to patients you have prescribed in the past month??

- ❑ Yes
- ❑ No

In your opinion, can insulin injection techniques affect the clinical outcomes of insulin therapy?

- ❑ Yes
- ❑ No

In your opinion, who should provide education related to Insulin Injection Technique to patients?

- ❑ Doctor
- ❑ Nurse
- ❑ Pharmacist
- ❑ Other healthcare workers

Do you use special guidelines in educating your patients about insulin injection?

- ❑ Yes
- ❑ No

Are you aware of the Indonesian Insulin Injection Technique Guidelines (PTMII) issued by the Forum for Injection Technique Indonesia?

- ❑ Yes
- ❑ No

Do you use PTMII as a reference in educating your patients about insulin injection techniques?

- ❑ Yes
- ❑ No

How often have you provided education on insulin injection techniques to your patients in the past month?

- ❑ Never
- ❑ 1-10
- ❑ 10-50
- ❑ > 50

If you use PTMII, how did you learn about this PTMII?

- ❑ I do not use PTMII
- ❑ Seminar/ symposium
- ❑ Website
- ❑ Books
- ❑ Information from PEDI or other health associations
- ❑ Information from pharmaceutical/medical device companies
- ❑ Others

If you use PTMII, of the several topics discussed in PTMII, which is the most important part in your opinion? (Choose a maximum of 3)

- ❑ I do not use PTMII
- ❑ General insulin therapy
- ❑ Insulin Injection Equipment
- ❑ Insulin Injection Techniques
- ❑ How to Use Insulin Vials & Syringes Correctly
- ❑ How to Use Insulin Pens and Pen Needles Correctly
- ❑ Special Attention to Insulin Injections
- ❑ Special Populations

Which of the following Insulin Injection Techniques using insulin pens and pen needles plays the most important role in insulin therapy?

- ❑ Pen needle length
- ❑ Pen needle gauge size
- ❑ Injection site selection
- ❑ Pinch technique
- ❑ Use of disposable needle for one injection

What can be done to better communicate PTMII to doctors or other healthcare workers?

QUESTIONNAIRE 2

KNOWLEDGE REGARDING INSULIN

Where do you most often recommend patients for self-administration of insulin?

- ❑ Upper arm
- ❑ Abdomen
- ❑ Thigh

Question	Agree	Disagree
1. Insulin must be injected at the same area and location		
2. Insulin absorption is influenced by the accuracy in choosing the insulin injection site		
3. The speed of insulin absorption is the same wherever it is injected		
4. The accuracy of insulin injection timing affects the results of blood sugar levels		
5. The injection time of fast-acting insulin analogue (Aspart, Glulisin, Lispro) with short-acting human insulin (Actrapid, Humulin-R) is the same		
6. Tense muscles during the insulin injection process can reduce pain		
7. Insulin injection in the abdominal area is very suitable for thin diabetes mellitus patients		
8. Insulin injection into intramuscular tissue can reduce pain compared to subcutaneous tissue		
9. Injection of fast-acting insulin analogue (Aspart, Glulisin, Lispro) in the thigh area is good for controlling blood sugar levels		
10. Rotation of insulin injections is needed to avoid lipodystrophy		
11. Before injecting insulin into thin diabetes mellitus patients, it is better to pinch the area to be injected to avoid injection intramuscular		
12. Rotation of insulin injections every week is done in one quadrant, following a clockwise direction, with a distance of 1 cm or 1 finger		
13. Rapid-acting insulin analogue (Aspart, Glulisin, Lispro) can be injected immediately after eating		
14. Insulin analogue injections are done 30 minutes after insulin is taken from the refrigerator		

15. Needles that are removed before 10 seconds after insulin is inserted can reduce the dose of insulin that is entered		
16. Insulin injected into intramuscular tissue will be absorbed faster than insulin injected into subcutaneous tissue		
17. The insulin injection site that is the fastest in insulin absorption is in the abdomen		
18. The most appropriate location for insulin injection is in the area experiencing lipohypertrophy		
19. The angle of insulin injection is generally perpendicular to the area to be injected		
20. Before injecting insulin at home, diabetes patients must always disinfect with alcohol		
