# Supplementary material for: Insulin Injection Technique Education and Associated Knowledge Factors Among Physicians: Cross-Sectional Survey Study
Source: JMIR Diabetes. 2025 Dec 8;10:e65359. doi: 10.2196/65359 (PMC12685283; doi:10.2196/65359)
Supplement: Checklist 1 [file diabetes-v10-e65359-s002.pdf]

Item Category	Checklist Item	Location
Design	Describe survey design	This is a cross-sectional online open survey
IRB approval and informed consent process	IRB approval	The ethical review for this study was approved by the ethics committee at Fatmawati Central General Hospital on February 26, 2021, numbered 10/KEP/II/2021.
	Informed consent	Informed consent was obtained before accessing the questionnaire.
	Data protection	All data were kept confidential and protected in online files, which can only be accessed by the investigators.
Development and pre-testing	Development and testing	The questionnaire was created by the research team at the Fatmawati Central General Hospital Integrated Diabetes Service Center, comprising 2 endocrinologists, 2 internists, and an epidemiologist. The questionnaire was assessed for usability, technical functionality, and reliability before its final version was distributed to the study participants. The questionnaire was pretested on 30 individuals not included in the main study (10 general practitioners, 8 internal medicine residents, 8 internists, and 4 endocrinologists), which yielded a KR-20 reliability coefficient of 0.70. The final version of the questionnaire

		was then distributed among groups.
Recruitment process and description of the sample having access to the questionnaire	Open survey versus closed survey	This is a cross-sectional online open survey
	Contact mode	The authors recruited potential participants (the target population was all physicians in Indonesia) by sending invitations to instant messaging (WhatsApp) groups for seminars/workshops/training, university alumni, hospitals, study/work societies, etc., using a convenience sampling method.
	Advertising the survey	The survey link was disseminated specifically to licensed physicians in Indonesia through professional networks, associations, and institutional mailing lists.
Survey administration	Web/E-mail	The authors recruited potential participants (the target population was all physicians in Indonesia) by sending invitations to instant messaging (WhatsApp) groups for seminars/workshops/training, university alumni, hospitals, study/work societies, etc., using a convenience sampling method.
	Context	Google forms
	Mandatory/voluntary	While anonymous and voluntary, this approach ensured that only qualified physicians participated in the study.
	Incentives	The subjects did not receive any compensation related to the study.

	Time/date	Data collection lasted for two weeks, from February 28th to March 14th, 2021
	Randomization of items or questionnaire	Not applied
	Adaptive questioning	Not applied
	Number of items	The questionnaire had 32 questions divided into 4 sections
	Number of screen (pages)	There were seven online pages of the questionnaire
	Completeness check	The completeness check was to ensure that the participants had completed the questionnaire completely.
	Review step	In the last page, the participants were also able to review and change the answer before submitting the results.
Response rates	Unique site visitor	Not applied
	View rate (Ratio of unique survey visitor/unique site visitors)	Not applied
	Participant rate (Ratio of unique visitors who agreed to participate/unique first survey page visitors)	Not applied
	Completion rate (Ratio of users who finished the survey/users who agreed to participate)	Not applied
Preventing multiple entries from the same individual	Cookies used	Not applied
	IP check	Not applied
	Log file analysis	Not applied
	Registration	Not applied
Analysis	Handling of incomplete questionnaire	The authors did not conduct data imputation or missing data analysis in this study.
	Questionnaire submitted with an atypical timestamp	Not applied
	Statistical correction	Not applied
